# Effects of endocannabinoids on feed intake, stress response and whole-body energy metabolism in dairy cows

**DOI:** 10.1038/s41598-021-02970-0

**Published:** 2021-12-08

**Authors:** Isabel van Ackern, Ramona Wulf, Dirk Dannenberger, Armin Tuchscherer, Björn Kuhla

**Affiliations:** 1 Research Institute for Farm Animal Biology (FBN), Institute of Nutritional Physiology ‘Oskar Kellner’, Wilhelm-Stahl-Allee 2, 18196 Dummerstorf, Germany; 2 Albrecht Daniel Thaer-Institute of Agricultural and Horticultural Sciences, Humboldt-Universität Zu Berlin, Invalidenstr. 42, 10099 Berlin, Germany; 3 Research Institute for Farm Animal Biology (FBN), Institute of Muscle Biology and Growth, Wilhelm-Stahl-Allee 2, 18196 Dummerstorf, Germany; 4 Research Institute for Farm Animal Biology (FBN), Institute of Genetics and Biometry, Wilhelm-Stahl-Allee 2, 18196 Dummerstorf, Germany

**Keywords:** Biochemistry, Physiology

## Abstract

Endocannabinoids, particularly anandamide (AEA) and 2-arachidonoylglycerol (2-AG), are instrumental in regulating energy homeostasis and stress response. However, little is known about the endocannabinoid system (ECS) in ruminants, although EC could improve dairy health and productivity, at least by increasing feed intake. In this study, we report if intraperitoneal (i.p.) AEA and 2-AG administration affects feed intake, whole-body macronutrient metabolism, isolation and restraint stress, and whether diet composition modulates circulating endocannabinoid concentrations in cows. Twenty Simmental cows in late lactation were fed a grass silage and a corn silage based diet. On each diet, cows received daily i.p. injections with either AEA (5 µg/kg; *n* = 7), 2-AG (2.5 µg/kg; *n* = 6) or saline (*n* = 7) for 8 days. Endocannabinoid administration for 5 days under free-ranging (non-stressed) conditions had no effect on feed intake or energy balance, but attenuated the stress-induced suppression of feed intake when housing changed to individual tie-stalls without social or tactile interaction. Endocannabinoids increased whole-body carbohydrate oxidation, reduced fat oxidation, and affected plasma non-esterified fatty acid concentrations and fatty acid contents of total lipids. There was no effect of endocannabinoids on plasma triglyceride concentrations or hepatic lipogenesis. Plasma AEA concentrations were not affected by diet, however, plasma 2-AG concentrations tended to be lower on the corn silage based diet. In conclusion, endocannabinoids attenuate stress-induced hypophagia, increase short-term feed intake and whole-body carbohydrate oxidation and decrease whole-body fat oxidation in cows.

## Introduction

The endocannabinoid system (ECS) is a complex network ubiquitously expressed throughout the body. The best studied endocannabinoids (EC) N-arachidonylethanolamide (anandamide; AEA) and 2-arachidonoylglyerol (2-AG) are synthesized from membrane phospholipids, with fat depots providing an important source for plasma EC^1^. Due to their amphiphilic character, EC can modulate the activity of many membrane proteins and activate the G protein-coupled cannabinoid receptors 1 and 2 (CB_1_ and CB_2_) or the G protein-coupled receptor 55 (GPR55), all of them expressed at the cell surface^2,3^. Primary enzymes degrading EC are fatty acid amide hydrolase (FAAH) for the breakdown of AEA and monoglyceride lipase (MGLL) for the degradation of 2-AG. Thus, FAAH and MGLL are involved in decreasing the EC tone, contributing to the short half-life of 2-AG and AEA of only a few minutes in mice^4,5^.

In rodents, a plethora of studies have shown that EC play an important role in the regulation of energy homeostasis^6^ involving the control of energy intake^7–9^ and energy expenditure^10^. The administration of AEA and 2-AG has been shown to stimulate feed intake of rats not only by acting at central but also peripheral sites. The injection of AEA or 2-AG into the brain^11,12^ as well as into the peritoneal cavity^13^ increased feed intake of rodents for a few hours. Peripherally administered EC may rapidly cross the blood–brain barrier^14^ or can activate vagal afferents in the periphery, thereby triggering signaling of the gut-brain axis^15–17^. The endogenous AEA and 2-AG tone in mice can be increased by diets supplemented with EC precursors such as arachidonic acid or linoleic acid^18,19^. By contrast, dietary lipids with higher content in n-3 polyunsaturated fatty acids (n-3 PUFA) can reduce the EC tone and diminish CB_1_ receptor activation in mice^20^.

In addition, EC are further involved in the regulation of the hypothalamic–pituitary–adrenal (HPA) axis^21^, which in turn affects not only energy metabolism but also stress response. Restraint stress has been demonstrated to decrease the AEA, but increase the 2-AG content in the amygdala of rats^22,23^. Moreover, pharmacological enhancement of EC signaling has been shown to attenuate the response to restraint stress in rodents by reducing corticosterone release^24,25^. Furthermore, EC support anabolic metabolism by promoting fat storage and decreasing energy expenditure^26^. More specifically, activation of CB_1_ stimulates lipogenesis in adipose cells^27^ and induces the expression of the lipogenic transcription factor sterol regulatory element-binding protein 1c (SREBP-1c) and its targets acetyl-CoA carboxylase-1 (ACC1) and fatty acid synthase (FAS) in liver^28^. However, knowledge about the involvement of the ECS in whole-body energy metabolism is mainly obtained from studies using CB_1_ antagonists (e.g. rimonabant, which is known to exert unwanted side effects) or CB_1_ KO mice^29,30^, but there is scarcity of studies using the natural endogenous receptor agonists.

As proposed by Myers, et al.^31^, activating the ECS in cows could provide the opportunity to improve dairy health and increase productivity by increasing feed intake and improving energy partitioning. Previous studies in periparturient dairy cows have shown the involvement of the ECS in adipose tissue metabolism, more specifically, an activation of the ECS in animals exhibiting high rates of lipolysis^32^. Furthermore, it has been shown that increased plasma AEA and 2-AG concentrations are directly associated with feed intake of dairy cows^33^, however, a direct cause-effect relationship has not been demonstrated yet. Also, feeding a ration above energetic requirements increased the expression of the CB_2_ receptor in bovine liver^34^, but whether the diet composition affects the EC tone, or how EC affect hepatic fat metabolism in ruminants is not known.

The aim of the present study was to evaluate the effect of i.p. administered AEA and 2-AG administration on isolation and restraint stress, feed intake, milk yield, whole-body macronutrient and hepatic fat metabolism in dairy cows, and to investigate if these effects are influenced by feeding a grass silage (GS) compared to a corn silage (CS) based diet. Given the fact that the n − 6/n − 3 ratio is typically higher in CS than GS and that increased dietary n − 3 PUFA reduce the EC tone, we hypothesized that the effect of EC administration would be more pronounced on the CS diet.

## Methods

### Animals and experimental design

Experiments were performed at the Research Institute for Farm Animal Biology (FBN), Dummerstorf, Germany and were in accordance with the ARRIVE guidelines (https://arriveguidelines.org), the German Animal Welfare Act and approved by the ethics committee of the State Government in Mecklenburg-Western Pomerania, Germany (Registration No. LALLF M-V 7221.3-1.1-041/18). Twenty non-pregnant Simmental cows from 1st to 10th lactation, in late lactation with an average milk yield of 15.6 ± 1.1 L per day and 263 ± 22 days in milk (DIM) were adapted to the experimental facilities for at least 20 days. The experiment was set up as a randomized block with a split plot of diet type (Supplementary Fig. S1), where animals entered the experimental trial in 5 blocks of four animals each. One block experiment lasted for 8 weeks and consisted of two 4-week feeding periods. Diets were fed in a cross over design, and the first 20 days of the second feeding period was considered the washout period. The cows were kept in a free-ranging barn and received two total mixed rations (TMR, Supplementary Table S1) based on either GS (low n − 6/n − 3 ratio) or CS (high n − 6/n − 3 ratio) with an average metabolizable energy (ME) of 10.7 ± 0.2 and 11.5 ± 0.1 MJ/kg of dry matter (DM), respectively. Troughs were filled at 05:00 a.m. and again at 05:00 p.m. Access to ad libitum intake was restricted between 05:00 a.m. and 08:00 a.m. to ensure a comparable start of feed intake. Milking took place twice daily (05:30 a.m. and 05:30 p.m.) and the milk yield was recorded. Body weight (BW) was automatically recorded when the cows passed through a scale after each milking and the metabolic BW (mBW = BW^0.75^) was calculated from a weekly mean. After balancing for mBW, lactation, milk yield and DIM, animals were allocated to 3 treatment groups: 2-AG (*n* = 6), AEA (*n* = 7) and NaCl (control, *n* = 7). Treatments were administered once-daily at 08:00 a.m. during the last 8 days of each feeding period, with cows receiving the same treatment during both feeding periods. An additional 9^th^ injection was given at the end of the second feeding period at 07:00 a.m. (day 9), approximately 1 h before slaughter of the NaCl-treated and approximately 2 to 3 h before slaughter of the EC-treated animals. The control group was injected 8 ml of a sterile 0.9% NaCl solution. 2-AG and AEA were administered at doses of 2.5 and 5 µg/kg BW, respectively, with a prime of twice the daily dose on day 1. The 2-AG and AEA solutions (pre-dissolved in ethanol) were purchased from Tocris (Bioscience, Bristol, UK) and diluted in 8 mL sterile 0.9% NaCl directly before administration. Doses in the low microgram/kg range were chosen because they were found to be most effective in dose response studies in rodents^9,35^ and exert no unwanted cannabimimetic side effects^36,37^. Intraperitoneal injections were administered into the right paralumbar fossa (1.20 mm × 80 mm, SUPRA, Vivomed, Geislingen, Germany). The i.p. administration route was chosen to allow absorption from the peritoneal cavity into systemic circulation, as well as to activate the splanchnic ECS and vagal afferents of the gut-brain-axis^15,38^. Immediately following the injection, cows were given free access to feed. Feed intake in the barn was constantly measured with a Roughage Intake Control system (RIC, Insentec B. V., Marknesse, Netherlands) and was documented as disappearance of feed from troughs, to determine individual feed intake.

### Indirect calorimetry

On day 6 of each treatment period, cows were transferred from the free- ranging barn into open-circuit respiration chambers and kept in individual tie-stalls^39^ to induce isolation and restraint stress. Cows were fitted with an indwelling jugular vein catheter (Cavafix Certo Splittocan 338, B. Braun Melsungen AG, Melsungen, Germany) connected to a 4-m extension line to allow for blood sampling from outside of the chamber without disruption of gas exchange measurements and animal behavior. After over-night equilibration, gas exchange measurement started on day 7 at 07:00 a.m. and lasted for 2 consecutive 24 h-periods at 15 °C. Intraperitoneal injections were administered at 08:00 a.m., and animals were fed immediately after injection and again at 05:00 p.m. for ad libitum intake. Milking in the chamber was performed at 06:00 a.m. and 04:00 p.m. Feed intake was measured as feed disappearance from trough by an electronic registration device (PAARI, Erfurt, Germany) every 6 min. The CO_2_ and CH_4_ concentrations were analyzed by infrared absorption (SIDOR, Sick AG, Reute, Germany) and O_2_ concentration was analyzed paramagnetically (SIDOR) in 6-min intervals. The airflow through the chamber (approximately 10 m^3^/h) was measured by a differential pressure type V cone flow meter (McCrometer, Hemet, CA). The mean CO_2_ recovery rate for all 4 chambers was 99.9%.

For the following calculations, data were used as mean of both 24 h-periods. Total CO_2_ production (CO_2 total_) is the sum of fermentative CO_2_ (CO_2 ferm_) and metabolic CO_2_ (CO_2 metab_). CO_2 ferm_ was estimated according to Chwalibog et al.^40^ as CO_2 ferm_ (L) = 1.7 × CH_4_ (L) in which 1.7 is constant for a variety of diet compositions^41^. Therefore, CO_2 metab_ was calculated by subtracting CO_2 ferm_ from CO_2 total_^42^. Net carbohydrate oxidation (COX) and net fat oxidation (FOX) were calculated by equations described by Frayn^43^:

$$\begin{aligned} {\text{COX }}\left( {\text{g}} \right) & = {4}.{\text{55CO}}_{{\text{2 metab}}} \,\,\left( {\text{L}} \right){-}{3}.{\text{21O}}_{{2}} \,\, \left( {\text{L}} \right){-}{2}.{\text{87N}}_{{\text{u}}} \,\,\left( {\text{g}} \right); \\ {\text{FOX }}\left( {\text{g}} \right) & = {1}.{\text{67O}}_{{2}} \,\, \left( {\text{L}} \right){-}{1}.{\text{67 CO}}_{{\text{2 metab}}} \,\, \left( {\text{L}} \right){-}{1}.{\text{92N}}_{{\text{u}}} \,\, \left( {\text{g}} \right). \\ \end{aligned}$$

The metabolic heat production (HP) was calculated according to Brouwer ^44^:

$${\text{HP }}\left( {{\text{kJ}}} \right) = {16}.{\text{18 O}}_{{2}} \,\, \left( {\text{L}} \right) + {5}.0{\text{2CO}}_{{\text{2 total}}} \,\, \left( {\text{L}} \right){-}{2}.{\text{17CH}}_{{4}} \,\, \left( {\text{L}} \right){-}{5}.{\text{99 N}}_{{\text{u}}} \,\, \left( {\text{g}} \right).$$

Urine N excretion (N_u_) was not measured but estimated to 100 g/d, although real N_u_ may vary from 75 to 150 g/d^45^, thereby accepting an error in HP, COX and FOX of less than 10%.

Indirect calorimetry data was normalized to mBW and to account for individual differences among animals changes of FOX, COX, and HP were calculated relative to the start of each measurement period (07:00 a.m.). The resulting ΔFOX/mBW, ΔCOX/mBW, and ΔHP/mBW data were evaluated in hourly intervals between 07:00 a.m. and 05:00 a.m. of the following morning. In addition, daily values of FOX, COX and HP were adjusted to DMI and mBW.

### Feed and milk analyses

Dry matter (DM) content of feed was determined weekly by drying pooled feed samples for 24 h at 60 °C followed by 4 h at 103 °C. Dry matter intake (DMI) was calculated from daily feed intake and weekly determined feed DM. During respiration chamber measurements, additional samples were taken for chemical analysis of nutrient composition. Chemical composition was analyzed by the accredited laboratory of Landwirtschaftliche Untersuchungs- und Forschungsanstalt der LMS Agrarberatung GmbH (LUFA, Rostock, Germany; Supplementary Table S1). The metabolizable energy (ME) content of the diet was calculated based on the German Society of Nutrition Physiology (GfE)^46^, and the ME intake (MEI) was calculated according to MEI (MJ of ME/d) = ME (MJ/kg of DM) × DMI.

Fatty acid composition of the diets was analyzed according to Kalbe et al.^47^, by using a modified method from Sukhija and Palmquist^48^ for direct fatty acid methylation. The extracts were subjected to gas chromatography (GC) analysis using a CP-Sil 88 CB column (100 m × 0.25 mm, Agilent, Santa Clara, CA, United States) in a PerkinElmer gas chromatograph CLARUS 680 with a flame ionization detector (FID; PerkinElmer Instruments, Shelton, United States). The detailed GC conditions were described by Dannenberger, et al.^49^ and average fatty acid composition of diets were shown in Supplementary Table S1.

Milk samples were pooled once a week from the evening and morning milking and analyzed for milk composition by infrared spectroscopy (MilkoScan; Foss GmbH, Hillerød, Denmark) at the State Inspection Association for Performance and Quality Testing Mecklenburg-Western Pomerania e.V. (LKV Güstrow, Germany). Additional samples from individual milking were taken between days 6 and 8 of the treatment period and analyzed using the same method by the Milk Testing Services North Rhine-Westphalia (LKV Krefeld, Germany). Milk composition was used to calculate the individual energy corrected milk yield (ECM) according to the GfE^50^: ECM (kg/d) = milk yield (kg/d) × ((1.05 + 0.38 × milk fat % + 0.21 × milk protein %)/3.28). Energy balance (EB) was estimated according to the GfE^50^: EB (MJ of ME/d) = MEI – (3.14 × ECM + 0.488 × mBW).

For analysis of DMI/mBW, EB, and ECM data were calculated as means during the pre-treatment period (PB, day − 5 to 0), treatment under normal, non-stressed housing conditions in the barn (TB, day 1 to 5) and treatment under stressed housing conditions in the respiration chamber (TC, day 7 and 8). Additionally, percent changes from PB to TB and TC were calculated.

### Blood sampling and analyses

To evaluate changes in fat metabolism, preprandial blood samples were collected on day 1 and day 9 at 07:00 a.m., each before EC administration in EDTA-containing tubes. Blood samples were immediately placed on ice and centrifuged at 1570 × *g* for 20 min at 4 °C, and obtained plasma was stored at − 80 °C. Plasma free, non-esterified fatty acid (NEFA), triglyceride (TG) and cholesterol concentrations were analyzed spectrophotometrically with a semi-automatic analyzer (ABX Pentra 400, HORIBA Medical, Kyoto, Japan) using the following kits: NEFA-HR 91797 (FUJIFILM Wako Chemicals Europe GmbH, Neuss, Germany), Triglycerides CP A11A01640 (HORIBA) and Cholesterol mono ChOD/PAP 900300 (mti-diagnostics GmbH, Idstein, Germany). The analysis of individual fatty acid concentrations in total plasma lipids was performed according to Dannenberger et al.^51^. Lipids were extracted by adding 1500 µL of plasma sample to a solution of chloroform/methanol (2:1, v/v) and internal standard (C19:0) at room temperature. Extracted lipids were methylated using sodium methylate and boron trifluoride methanol, and major fatty acids detected are listed in Supplementary Table S4. For analysis of individual changes in plasma lipid metabolites percent changes were calculated from pre-treatment to after 8 days of treatment.

For the analysis of plasma EC concentrations, plasma samples collected on day 8 prior to morning feeding and treatment were analyzed for AEA and 2-AG using the cross-validated method as described by Zoerner et al.^52^. Briefly, 18-µL of an ethanolic solution of the internal standards d5-2-AG (53.3 pg/µL) and d4-AEA (50 pg/µL) were added to the thawed plasma samples and incubated for 15 min on ice. Solvent extraction was performed by adding toluene (1 mL) and by shaking twice in a Precellys 24 Dual Homogenisator at 5000 rpm for 20 s. Phase separation was achieved by centrifugation (4655 × *g*, 4 °C, 5 min). The upper organic phase was evaporated at room temperature under nitrogen. To the residue a 40-µL aliquot of water–methanol (1:3, v/v) was added and mixed by vortexing for 10 s. Analyses were performed on a Waters ACQUITY UPLC-MS/MS system with a tandem quadruple mass spectrometer XEVO TQ MS (Waters, Milford, MA, USA). Separation of analytes was carried out on a Waters ACQUITY BEH C18 column (100 mm × 2.1 mm i.d., 1.7 µm particle size) at 60 °C.

### Liver tissue, RNA extraction and RT-qPCR

On day 9 of EC treatment of the second feeding period, cows were stunned with a captive bolt gun and immediate exsanguinated at the institute’s slaughterhouse. Liver samples were taken and immediately snap frozen in liquid nitrogen and stored at − 80 °C until further analysis. Tissue samples were ground under liquid N. RNA was extracted from 20 to 25 mg tissue powder with innuPREP RNA Mini Kit 2.0 (Analytik Jena AG, Jena, Germany) and residual DNA was removed with innuPREP DNase I Digest Kit (Analytik Jena AG). RNA concentrations were quantified spectrophotometrically on a NanoPhotometer (Implen GmbH, Munich, Germany). RNA quality was assessed using an Agilent 2100 Bioanalyzer (Agilent, Santa Clara, CA, USA), yielding RNA integrity number (RIN) factors between 7.0 and 8.7 (mean 7.7 ± 0.13). For cDNA synthesis, 750 ng total RNA was reverse transcripted (SensiFAST cDNA Synthesis Kit, Bioline, London, UK) using a Thermocycler (peqstar 96 × HPL, VWR International, Pennsylvania, USA). Transcriptional expression was quantified by real-time PCR (qPCR) using the following primers (Supplementary Table S2). qPCR was performed on a LightCycler 2.0 (Roche, Basel, Switzerland) with SensiFAST SYBR No-ROX Kit (Bioline) with 2 µL of cDNA. Each cDNA sample was analyzed in duplicate. Primer products were sequenced and the correct sequence was confirmed. The efficiency of amplification was calculated using LinRegPCR software, version 2014.4 (Academic Medical Centre, Amsterdam, Netherlands), yielding efficiency values between 1.84 and 1.92 (Supplementary Table S2). Data were quantified by qbasePlus software (Biogazelle, Gent, Belgium) and normalized to the reference genes eukaryotic translation initiation factor 3 subunit K (*EIE3K*) and peptidylprolyl isomerase A (*PPIA*).

### Statistical analyses

Data were analyzed with mixed models (PROC MIXED) and repeated measures using SAS software (version 9.4, SAS Institute Inc., Cary, NC, USA). Unless otherwise stated, the random effects of all models were block and block by period interaction and a compound symmetry covariance structure was used for all data. Denominator degrees of freedom were estimated by using the Satterthwaite option in the MODEL statement and paired differences were determined using the SLICE option in PROC MIXED.

The DMI/mBW, EB, ECM, and percent changes in DMI/mBW, EB, ECM were analyzed with the fixed effects of treatment, housing, diet and all associated interactions. Housing was used in the repeated statement to account for repeated measures and the subject was defined as cow by period interaction. The ΔFOX/mBW, ΔCOX/mBW, and ΔHP/mBW were analyzed with the fixed effects of treatment, time interval, diet and all associated interactions. Time interval was used in the repeated statement and the subject was defined as cow by period interaction.

The FOX, COX and HP values adjusted to DMI and mBW as well as the percent changes in plasma lipid metabolites data were analyzed with the fixed effects of treatment, diet and their interaction. Period was used in the repeated statement and the subject was defined as cow. The mRNA abundances of hepatic genes were analyzed with the fixed effect of treatment and the random effect of block.

Plasma AEA and 2-AG concentrations were analyzed using the *t*-test procedure of SAS. Data were tested for a normal distribution with a Shapiro–Wilk test and transformed using the Johnson transformation prior to testing if necessary. Transformed data are noted in the table. Calculation of Pearson correlation between dietary fatty acids and EC concentrations was performed by using the CORR procedure of SAS. Significance was declared at *P* ≤ 0.05 and tendencies were declared at 0.05 < *P* ≤ 0.10. Results are presented as LSM ± SE unless stated otherwise.

## Results

### Dry matter intake, energy balance and energy corrected milk yield

**Figure 1:**
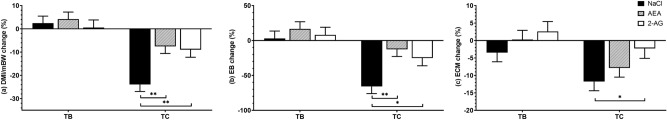
Percent changes (%) in dry matter intake (DMI) per mBW (**a**), energy balance (EB) (**b**) and energy corrected milk yield (ECM) (c) after i.p. injections with NaCl (n = 7), AEA (n = 7) or 2-AG (n = 6) under free-ranging, non-stressed conditions in the barn (TB), and under stressed conditions in the respiration chamber (TC) relative to pre-treatment. Change of housing to TC significantly decreased DMI/mBW, EB and ECM in all treatment groups (*P* < 0.05). Significant pairwise effects within-housing were detected only under stressed conditions in the respiration chamber (TC). The AEA and 2-AG treatment significantly attenuated the stess-induced decrease in DMI/mBW and EB (*P* < 0.05) and 2-AG treatment significantly attenuated the stess-induced decrease in ECM (*P* < 0.05). Graphs are presented as highest level of significant interaction, associated *P*-values can be found in Supplementary Table S3. Within-housing differences are indicated by * *P* < 0.05 and ** *P* < 0.01 (Tukey–Kramer).

DMI normalized to mBW, EB and ECM did not differ between groups pre-treatment (PB) (Supplementary Fig. S2A) and were higher on the CS than GS diet (*P* < 0.05). However, the diet had no effect on the treatment and there was no three-way interaction of diet, treatment and housing. The analysis of inidvidual changes in DMI/mBW revealed that the i.p. EC treatment under normal, non-stressed conditions in the barn (TB) for 5 days had no effect (Fig. 1a). Continuation of treatments but housing change to respiration chambers (TC) inducing isolation and restraint stress decreased DMI/mBW in all groups compared to PB (*P* < 0.001). However, the reduction in DMI/mBW from PB to TC was only 8.6 ± 3.6% with AEA and 9.6 ± 3.8% with 2-AG treatment, and was significantly less (*P* < 0.01) than in the control group (22.4 ± 3.6%, Fig. 1a). Consistent with changes in DMI/mBW, i.p. EC treatment TB had no effect on EB (Fig. 1b) and when cows were exposed to the stressful environment, EB declined (*P* < 0.001). The decline in EB was only 15.8 ± 12.6% with AEA, 28.4 ± 13.0% with 2-AG, but 60.0 ± 12.6% in the control group (*P* < 0.05, Fig. 1b). Equally, ECM was not affected by i.p. EC treatment TB and declined with change of housing to TC (*P* < 0.001; Fig. 1c). The reduction in ECM from PB to TC was 7.8 ± 3.0% with AEA, 2.5 ± 3.1% with 2-AG, but 11.7 ± 3.0% in the control group. The reduction in the control group was significantly greater than in the 2-AG group (*P* < 0.05).

### Plasma lipids

**Figure 2:**
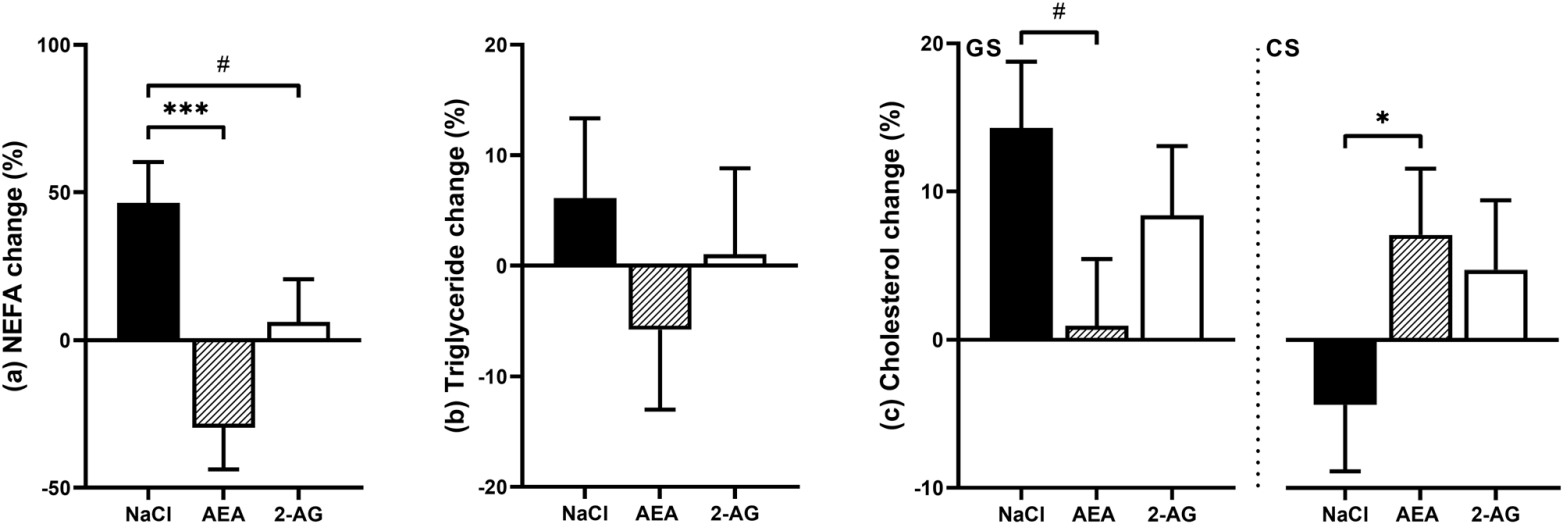
Percent changes (%) in plasma NEFA (**a**), triglyceride (TG) (**b**) and cholesterol (**c**) concentrations after i.p. injections of NaCl (n = 7), AEA (n = 7) or 2-AG (n = 6) for 8 days relative to pre-treatment. Plasma NEFA concentatrions significantly decreased after 8 days of treatment with AEA compared to the control group (*P* < 0.001) and tended to increase to a lesser extend with 2-AG treatment compared to the control group (*P* = 0.05). No changes were detected for percent changes in plasma TG. Plasma cholesterol percent changes differed in the control group between diets, as indicated by the treatment x diet interaction (*P* < 0.05). Graphs are presented as highest level of significant interaction, associated *P*-values can be found in Supplementary Table S3. Treatment differences are indicated by # < 0.1, * *P* < 0.05 and *** *P* < 0.001 (Tukey–Kramer).

The analysis of plasma lipid concentrations revealed some differences among animals before EC treatment, therefore, we analyzed the inidvidual changes from PB to TC. The percent changes in plasma NEFA concentrations were different between treatment groups (*P* < 0.001) (Fig. 2a). While plasma NEFA concentrations increased by 46.4 ± 13.8% in the control group and by 6.3 ± 14.5% in the 2-AG group, they decreased by 29.6 ± 14.2% in the AEA group. Resulting in a significant difference in the control group compared to the AEA group (*P* < 0.001) and a tendency to be different to the 2-AG group (*P* = 0.05). EC treatment had no effect on percent changes in plasma TG concentrations (Fig. 2b). However, TG concentration tended to be different between diets, with higher levels on the GS compared to the CS diet (10.7 vs − 9.73%; *P* = 0.06). Percent changes in plasma cholesterol concentrations differed in the control group between diets, as indicated by the treatment x diet interaction (*P* < 0.05) (Fig. 2c).

Among total lipids, the plasma palmitic acid (C16:0) content increased in the control group but declined in the AEA and 2-AG groups on both diets (*P* < 0.01; Supplementary Table S4). The oleic acid (C18:1*c*9) content also increased in the control group but remained unchanged in EC-treated animals on the GS diet and declined in EC-treated animals on the CS diet (*P* < 0.01). Palmitoleic acid (C16:1) content was affected by treatment (*P* < 0.05), with a greater decrease in the AEA and 2-AG compared to the control group. In contrast, linoleic acid (C18:2n6) was (*P* < 0.01) and stearic acid (C18:0) tended to be (*P* = 0.09) reduced in NaCl but increased or unchanged in EC-treated cows (Supplementary Table S4). Plasma arachidonic acid (C20:4n6) contents were not different between treatment groups. However, the contents of C20:4n6, C20:2n6, C20:3n6 and C22:4n6 tended to increase on the CS diet (*P* < 0.1). Percent changes in the plasma n − 6/n − 3 ratio tended to be different between treatment groups (*P* = 0.05) (Supplementary Table S4).

### Whole body energy metabolism

**Figure 3:**
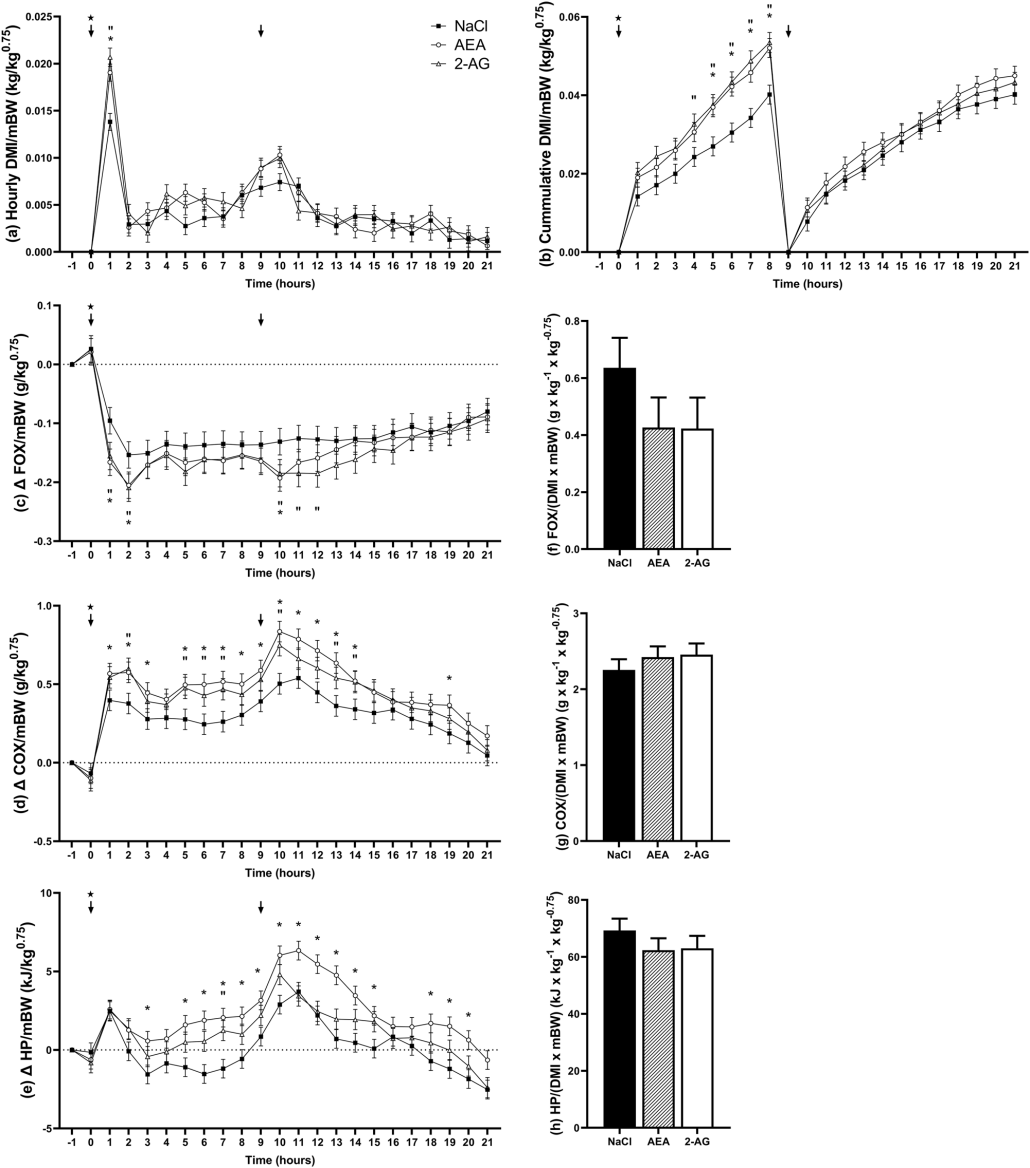
Hourly dry matter intake (DMI) per mBW (**a**), cumulative DMI/mBW (**b**), changes in fat oxidation per mBW (Δ FOX/mBW) (**c**), changes in carbohydrate oxidation per mBW (Δ COX/mBW) (**d**), changes in heat production per mBW (Δ HP/mBW) (**e**) and daily fat oxidation (FOX) (**f**), carbohydrate oxidation (COX) (**g**) and heat production (HP) (**h**), each normalized to dry matter intake (DMI) and metabolic bodyweight (mBW). Cows were intraperitoneally injected with either NaCl (n = 7), AEA (n = 7) or 2-AG (n = 6). Feed was withheld from 07:00 to 08:00 a.m. The star indicates the i.p. injection (08:00 a.m.), the arrow indicates feeding at 08:00 a.m. and 05:00 p.m. Graphs are presented as highest level of significant interaction, associated *P*-values can be found in Supplementary Table S3. Treatment differences between the control and AEA group are indicated by * (*P* < 0.05; Tukey–Kramer), and differences between the control and 2-AG group are indicated by “(*P* < 0.05; Tukey–Kramer).

Next, we examined short-term effects of EC on feed intake and whole body energy metabolism. The BW, body condition score and back fat thickness did not differ between groups (data not shown). DMI/mBW was not different between the GS and CS diet (Supplementary Table S3). Hourly DMI/mBW changed over time (*P* < 0.001) and differed between treatment groups (*P* < 0.05; Fig. 3a). Specifically, within the 1^st^ h after injection, i.p. AEA and 2-AG administration resulted in a 33.8 and 49.7% higher DMI/mBW compared to the control group (*P* < 0.001). Subsequently, hourly DMI/mBW was not significantly different between groups. Cumulative DMI/mBW increased over time (*P* < 0.001) and tended to be differed between treatment groups (*P* = 0.05; Fig. 3b). Within 8 h after i.p. injection and morning feeding, AEA and 2-AG treatment resulted in a 29.6 and 33.0% increase in cumulative DMI/mBW compared to the control group (*P* < 0.05). Effects of the EC treatments were no longer observed after the afternoon feeding at 05:00 p.m. Fat oxidation (FOX) ranged from -2.0 to 14.7 g per 6 min on the GS and from -4.6 to 10.2 g per 6 min on the CS diet (data not shown). The Δ FOX/mBW varied over time (*P* < 0.001; Fig. 3c) and was lower on the CS than GS diet (− 0.16 vs 0.09 g x kg^-1^ × kg^-0.75^; *P* < 0.05). AEA treatment resulted in a greater decrease in Δ FOX in the first 2 and 10th h after injection and was significantly lower than in the control group (*P* < 0.05). Likewise, 2-AG treatment resulted in a greater decrease in Δ FOX in the first 2 h and 10 to 12 h after injection and was significantly lower than in the control group (*P* < 0.05; Fig. 3c). Carbohydrate oxidation (COX) ranged from − 4.8 to 47.1 g per 6 min on the GS and from 3.9 to 52.2 g per 6 min on the CS diet (data not shown). Δ COX/mBW varied over time (*P* < 0.001) and was different between treatment groups (*P* < 0.01; Fig. 3d). Specifically, i.p. EC injection resulted greater increase in Δ COX/mBW with AEA and 2-AG treatment compared to control treatment (Fig. 3d). The increase in Δ COX/mBW was significantly higher with AEA treatment 1 to 3, 5 to 14 and 19 h and with 2-AG treatment 2, 5 to 7, 10 and 13 to 14 h after injection relative to the control (*P* < 0.05). Metabolic heat production (HP) normalized to mBW ranged from 380.4 to 740.7 kJ per 6 min on the GS and from 398.5 to 839.4 kJ per 6 min on the CS diet (data not shown). Δ HP/mBW was greater on the GS than CS diet (2.11 vs 0.02 kJ × kg^−1^ × kg^−0.75^; *P* < 0.001), changed over time (*P* < 0.001; Fig. 3e) and was different between treatment groups (*P* < 0.01; Fig. 3e). The i.p. AEA and 2-AG injections resulted in a greater increase in Δ HP/mBW. Specifically, the increase was significantly higher 3, 5 to 15 and 18 to 20 h after AEA treatment and 7 h after 2-AG treatment relative to the control (*P* < 0.05).

To account for the difference in feed intake among groups during indirect calorimetry, daily FOX, COX and HP were additionally normalized to individual DMI. FOX per mBW and DMI tended to be greater on the GS than the CS diet (0.66 vs 0.33 g × kg^-1^ × kg^−0.75^; *P* = 0.09). Double normalized FOX tended to be different between treatment groups (*P* = 0.08; Fig. 3f) and was 32.9% lower with AEA (*P* = 0.11) and 33.5% lower 2-AG (*P* = 0.12) compared to the control group. Double normalized COX tended to be higher on the CS than the GS diet (2.67 vs 2.08 g × kg^−1^ × kg^−0.75^; *P* = 0.05), but was not different between treatment groups (Fig. 3g). Double normalized metabolic HP was neither different between diets nor treatment groups (Fig. 3h).

### Transcription of Hepatic Genes

The analysis of the mRNA abundance of hepatic genes involved in the ECS (*CNR1, FAAH, MGLL, GPR55*) and fat metabolism (*ACAA2, ACACA, CPT1A, DGAT1 and 2, HADH, PLAAT5, PPARA and SREBF1*) revealed no differences between treatment groups (Table 1).

**Table 1:** Relative mRNA abundances of hepatic genes involved in endocannabinoid and fat metabolism.

Gene	NaCl	AEA	2AG	*P*-value
				Treatment
ACAA2	0.99 ± 0.06	0.99 ± 0.06	1.01 ± 0.06	0.96
ACACA	0.82 ± 0.19	1.25 ± 0.19	1.14 ± 0.21	0.29
CNR1	1.38 ± 0.22	0.96 ± 0.22	1.05 ± 0.24	0.39
CPT1A	1.09 ± 0.10	0.94 ± 0.10	1.04 ± 0.11	0.43
DGAT1	1.13 ± 0.25	0.92 ± 0.25	1.38 ± 0.27	0.47
DGAT2	1.21 ± 0.25	1.04 ± 0.25	1.19 ± 0.27	0.87
FAAH	1.13 ± 0.08	0.96 ± 0.08	0.97 ± 0.09	0.23
GRP55	1.12 ± 0.19	0.97 ± 0.19	1.20 ± 0.20	0.70
HADH	1.04 ± 0.06	0.96 ± 0.06	1.03 ± 0.07	0.61
PLAAT5	1.38 ± 0.42	1.31 ± 0.42	1.36 ± 0.45	0.99
MGLL	0.97 ± 0.16	1.16 ± 0.16	1.01 ± 0.17	0.65
PPARA	1.09 ± 0.19	1.32 ± 0.19	0.87 ± 0.21	0.31
SREBF1	1.13 ± 0.11	1.11 ± 0.11	0.86 ± 0.12	0.22

Liver tissue samples were obtained from cows treated with NaCl (*n* = 7), AEA (*n* = 7) or 2AG (*n* = 6) for 9 days.

ACAA2, acetyl-COA acyltransferase 2; ACACA, acetyl-CoA carboxylase alpha; CNR1, cannabinoid receptor 1; CPT1A, carnitine palmitoyltransferase 1A; DGAT1/ 2, diacylglycerol O-acyltransferase 1/ 2; FAAH, fatty acid amide hydrolase; GPR55, G protein-coupled receptor 55; HADH, hydroxyacyl-CoA dehydrogenase; PLAAT5, phospholipase A and acyltransferase 5; MGLL, monoglyceride lipase; PPARA, peroxisome proliferator activated receptor alpha; SREBF1, sterol regulatory element binding transcription factor 1.

### Plasma endocannabinoid concentrations

We next examined if the diet composition affected the basal plasma EC concentrations. As shown in Table 2, pre-treatment plasma AEA concentration of all cows was not affected by diet composition, however, plasma 2-AG concentrations tended to be 36% lower when animals were fed the CS diet (*P* < 0.10). There were no significant correlations between dietary fatty acids and plasma EC concentrations (data not shown).

**Table 2:** Plasma anandamide (AEA) and 2-arachidonoylglyerol (2-AG) concentrations (nM) of cows (*n* = 20) after ad libitum feeding of a grass silage (GS) and corn silage (CS) based diet for 27 days.

	GS	CS	*P*-value
			Diet
AEA (nM)^1^	0.13 ± 0.01	0.12 ± 0.01	0.53
2-AG (nM)^1^	50.0 ± 9.46	31.9 ± 5.67	< 0.10

Data presented as means ± SEM.

^1^Data transformed using the Johnson transformation for statistical analysis and back-transformed for interpretation.

## Discussion

### Effect of endocannabinoids on dry matter intake, energy balance, and isolation and restraint stress

Many studies have shown that EC regulate feed intake in rodents^7,13,35^. To the best of our knowledge, this is the first study describing the effects of i.p. administered AEA and 2-AG on feed intake and energy metabolism in lactating dairy cows. We found that administration of EC for 5 days did not alter daily feed intake when animals were kept under free ranging (non-stressed) conditions in a barn. The lack of feed intake response may be due to a too short administration period of 5 days, a high diurnal temperature variability in the barn, social competition over preferred feeding troughs, or because short-term changes in feed intake could not be detected on the daily measurement basis (see below). With change of housing and induction of restraint stress, DMI/mBW decreased in all groups consistent with the stress-induced hypophagia in rodents and humans^53,54^. In fact, isolation of cows without social and tactile interaction has been shown to induce stress, increasing plasma cortisol and decreasing feed intake^55,56^. Furthermore, stress is exacerbated by confinement and tethering^55,57^. Another reason for the decline in feed intake is the lower energy requirement for physical activity when animals are tie-stalled. However, the decline in feed intake with housing change was significantly less with EC treatments, indicating that AEA and 2-AG administration improved habituation and attenuated the stress response. Activation of the ECS by administering the CB_1_ agonist CP55940 or the FAAH inhibitor URB597 has shown to inhibit the restraint-induced activation of the HPA axis in mice^24^. Furthermore, CP55940 and URB597 blocked the restraint stress-induced decrease in sucrose consumption of mice^58^ and ameliorated stress^59^. Although we did not measure EC when cows were kept under normal, non-stressed housing conditions, plasma 2-AG concentrations were approximately 20- to 30-fold higher during isolation and restraint stress than basal levels of cows housed in a free-ranging, non-stressed environment^33^, further underscoring the involvement of EC in response to stress. Energy balance and ECM also decreased along with housing and stress level change which can be attributed to the decrease in feed intake. Furthermore, the decline in energy balance was significantly less with EC treatment, thus the extent of energy balance reduction can be ameliorated by EC treatment preventing an excessive decline in energy intake.

Additionally to a stress-protective role, AEA and 2-AG administration also increased feed intake during indirect calorimetry in the short-term. The 6-min recordings showed that immediately after AEA and 2-AG injection at dosages of 5 and 2.5 µg/kg, respectively, feed intake was higher than in controls for the first hour after injection only. This short-term response may explain why we did not observe differences in daily feed intake measured in the barn. The short-term effect could be due to the observed short half-life of AEA and 2-AG of only a few minutes in heparinized mouse blood^5^. Because the half-life of 2-AG was higher in human plasma compared to rat plasma, a different half-life between various mammals can be assumed^60^. The specific half-life of AEA and 2-AG in cows is not known and needs to be determined in future studies. However, plasma AEA concentrations of the cows studied were 2.5-fold increased 2.5 h after i.p. administration of 5 µg AEA/kg BW^61^. This finding indicates that AEA accounts for the increase in feed intake despite of its relative short half-life and further suggests that the half-life of AEA in cows is presumably not as short as it is in mice. A short-term (1 to 3 h) increase in feed intake was also observed after subcutaneous administration of 0.5 to 10 mg/kg AEA to rats^7^. However, other studies performed in mice reported that AEA^35^ and 2-AG^9^ each administered i.p. at 0.001 mg/kg, increased cumulative feed intake over 7 or 14 days, respectively. Higher dosages tested by these authors, e.g. 0.7 and 4 mg/kg AEA^35^, or 0.01 and 0.1 mg/kg 2-AG^9^ had no long-term effect on cumulative feed intake in mice. Future studies are needed to determine if higher EC dosages or multiple injections per day would increase feed intake also in the long-term, but care must be taken of the risk of unwanted cannabimimetic side effects e.g. hypomobility^36,37^.

### Effect of endocannabinoids on plasma lipids

The trend for the lower DMI-normalized FOX after EC treatment was accompanied by a reduction in plasma NEFA concentrations, at least for AEA-treated animals, whereas they increased in control animals. Plasma NEFA concentrations reflect body fat mobilization and function as a quantitative marker for lipolysis in dairy cows^62,63^. Therefore, it can be assumed that animals of the control group, showing an increase in plasma NEFA concentrations, mobilized some body fat, likely because of stress-induced hyophagia in the respiration chamber. Furthermore, the reduction in plasma NEFA concentrations in AEA-treated animals and the only minimal increase in 2-AG-treated animals might indicate that EC inhibited lipolysis in these animals despite a slight reduction in feed intake. Similarly, it has been shown that EC signaling activates lipogenic mechanisms^64^, e.g. CB_1_ activation enhanced lipogenesis in primary adipocyte cultures^27^. However, we did not measure lipolytic activity.

Total plasma fatty acids in cows can be predictive for lipid classes. While C16:0, C18:0 and C18:1c9 are the major fatty acids of the free fatty acid fraction or bound in TG, C18:2n6 is the predominant fatty acid bound in cholesterol esters and phospholipids^65^. Besides, plasma phospholipids may also contain substantial amounts of C16:0 and C18:0^65^. Our results of decreasing levels of C16:0, C16:1 and C18:1c9 after AEA and 2-AG administration for 8 days may indicate that EC facilitate the flux of free fatty acids to adipocytes for TG synthesis as described earlier^64,66^. However, plasma TG concentrations were found not different between control and EC groups. This finding is surprising as it has been reported that pharmacological stimulation of EC signaling increases plasma TG and cholesterol concentrations in mice^67^. Presumably, hypertriglyceridemia after EC treatment could not be observed in dairy cows as TG were excreted with milk, but this assumption needs further experimental approval. Plasma cholesterol concentrations increased in EC treated animals and in NaCl treated cows on the GS diet, however, they decreased in the control group on the CS diet. These results are in parallel with the stimulatory effect of EC on plasma cholesterol observed in mice^67^, suggesting reduced plasma lipoprotein clearance and accumulation of plasma lipoproteins in both mice and CS-fed cows. Plasma C18:2n6 decreased in control animals, while EC administration prevented this decrease, which is likely due to the lower feed intake of animals in the control group compared to EC treatment.

### Effect of endocannabinoids on whole-body energy metabolisms

Previous studies reporting on ECS involvement in whole-body energy metabolism mainly draw conclusions by investigating the role of CB_1_ antagonists or CB_1_ KO mice^29,30^. To the best of our knowledge, this is the first study describing the direct effects of i.p. administered AEA and 2-AG on whole-body energy metabolism.

Immediately after i.p. injections and morning feeding, whole body fat oxidation decreased while oxidation of carbohydrates increased in all groups, which is due to the high carbohydrate content in feed. An inverse relationship between COX and FOX, and between FOX and feed intake but a positive relationship between COX and feed intake has been reported earlier for dairy cows^42^. Accordingly, the higher feed intake after AEA and 2-AG treatment was accompanied by a higher COX, not only after the morning feeding but also in the afternoon. The longer-lasting effect in higher COX can be explained by the fact that more feed ingested with the morning feeding requires longer fermentation and digestion time which in turn delay metabolic CO_2_ production from dietary carbohydrates in post-absorptive metabolic processes. In parallel to the short-term response in feed intake after EC injection, the reduction in FOX was most pronounced in the first two hours after injection. Both FOX and COX contribute to total metabolic HP since the portion of heat produced from COX is higher than from FOX, and since COX and feed intake were higher in EC- compared to NaCl-treated cows, the resulting metabolic HP was higher in AEA and 2-AG treated animals.

To account for the differences in feed and energy intake between treatment groups and to examine whether changes were limited to a feed intake-related effect, daily FOX, COX and metabolic HP were additionally normalized to DMI. The analysis revealed that double normalized FOX tended to be lower in the AEA and 2-AG compared to the control group, suggesting that effects extend beyond differences in feed intake and that AEA and 2-AG suppress whole-body fat catabolism and/or increase lipogenesis. These findings support earlier indirect calorimetry studies reporting that the CB_1_ antagonist rimonabant increased energy expenditure in rats by increasing fat oxidation^29,30^. From these studies and since endocannabinoids were reported to favor anabolic processes^26,68^, we expected double normalized metabolic HP to be reduced in the AEA and 2-AG compared to the control group, but the EC effect could not be tested significantly lower. Apparently, the numeric increase in DMI-normalized COX overrides the tending FOX decrease induced by EC.

### Effect of endocannabinoids on hepatic gene expression

The expression of hepatic genes involved in the ECS and fat metabolism were not different between treatment groups. Our results are in contrast to studies performed in mice, where CB_1_ activation increased de novo fatty acid synthesis and increased hepatic gene expression of genes involved in lipogenesis, such as SREBP-1, ACC1 and FAS^28^. In contrast to monogastric species, the bovine liver is only marginally capable to perform de novo fat synthesis because of limited ACC abundance, whereas adipose tissue accounts for over 92% of whole-body fatty acid synthesis^69^. The lack of expression differences between treatment groups may also be due to the fact that i.p. administered EC had no direct effect on liver, or because of the high inter-individual variation in gene expression.

### Effect of diets on plasma endocannabinoid concentrations

Recent studies in mice have shown that feeding an elevated dietary linoleic acid (C18:2n6) content increases the AEA and 2-AG concentrations in brain and liver tissue^18,70^. The linoleic acid content of the diets tested in the present study was 12.9% higher in the CS than GS diet. Although we found numerically higher plasma C18:2n6 content in animals fed the CS diet (data not shown), plasma AEA concentrations were comparable between, and 2-AG plasma concentrations were even lower with CS feeding. Presumably, the 30% difference in the dietary linoleic acid content was not large enough to induce differences in plasma EC concentrations. This conclusion is supported by earlier studies reporting approximately twofold higher tissue EC levels with feeding an eightfold higher linoleic acid content^18,70^. However, the tending lower plasma 2-AG concentrations with CS feeding might be explained by the higher content n-3 PUFA, known to reduce the EC tone^20^, yet it remains questionable why this difference is not observed for AEA. Besides the different amounts of some constitutes in the rations, a major reason might be the lower energy intake of the animals on the GS diet, as the reduction of energy intake has been shown to stimulate 2-AG biosynthesis^71^.

## Conclusion

The present study demonstrates that i.p. administration of AEA and 2-AG reduces the stress-induced suppression of feed intake and stimulates feed intake in the short-term. As a consequence of the latter, both EC increase whole-body carbohydrate oxidation and metabolic heat production. EC administration reduces whole-body fat oxidation exceeding differences in feed intake, suggesting that AEA and 2-AG suppress whole-body fat catabolism and/or support lipogenesis. However, once daily EC administration do not affect plasma TG concentrations, hepatic lipogenesis and hepatic fatty acid oxidation in dairy cows. Future studies should also determine if higher EC dosages, multiple injections per day or continuous EC infusion would alter feed intake and metabolism in the long-term. Finally, feeding a corn silage based diet with a 30% higher linoleic acid content than grass silage did not increase plasma EC concentrations, thus more research is required to elucidate how diet formulation can modulate the EC tone in ruminants.

## Supplementary Information

Supplementary Information.

## Data Availability

All data generated and analyzed are available from the corresponding author on request.
