# Supplementary material for: Effects of endocannabinoids on feed intake, stress response and whole-body energy metabolism in dairy cows
Source: Sci Rep. 2021 Dec 8;11:23657. doi: 10.1038/s41598-021-02970-0 (PMC8655048; doi:10.1038/s41598-021-02970-0)
Supplement: Supplementary file 1 — Supplementary Information. [file 41598_2021_2970_MOESM1_ESM.docx]

**
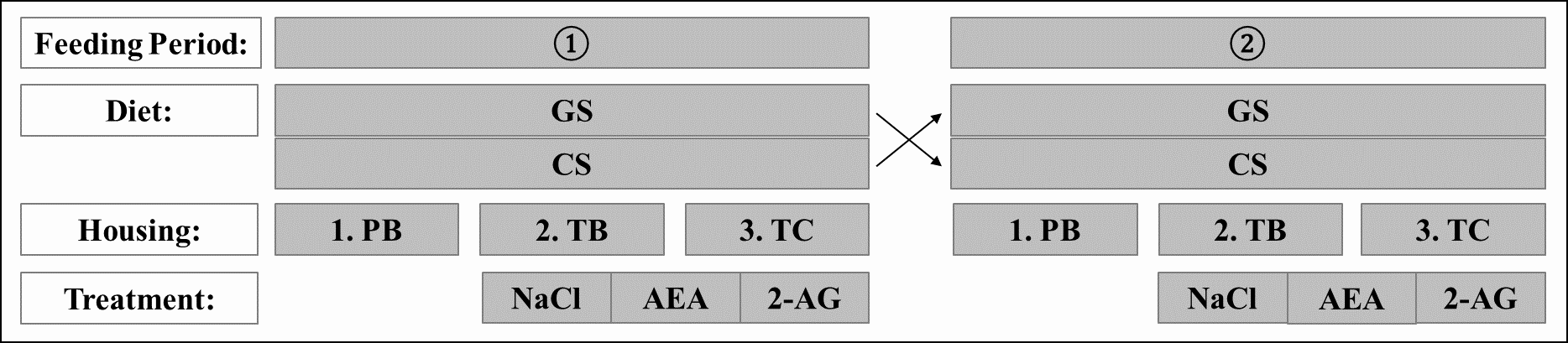
**

**Supplementary Figure S1.** Experimental design

Diet: GS grass silage based TMR; CS corn silage based TMR

Housing: PB pretreatment, barn; TB treatment, barn; TC treatment, respiration chamber

Treatment: NaCl saline; AEA anandamide; 2-AG 2-arachidonoylglycerol; with each cow receiving the same treatment in each period


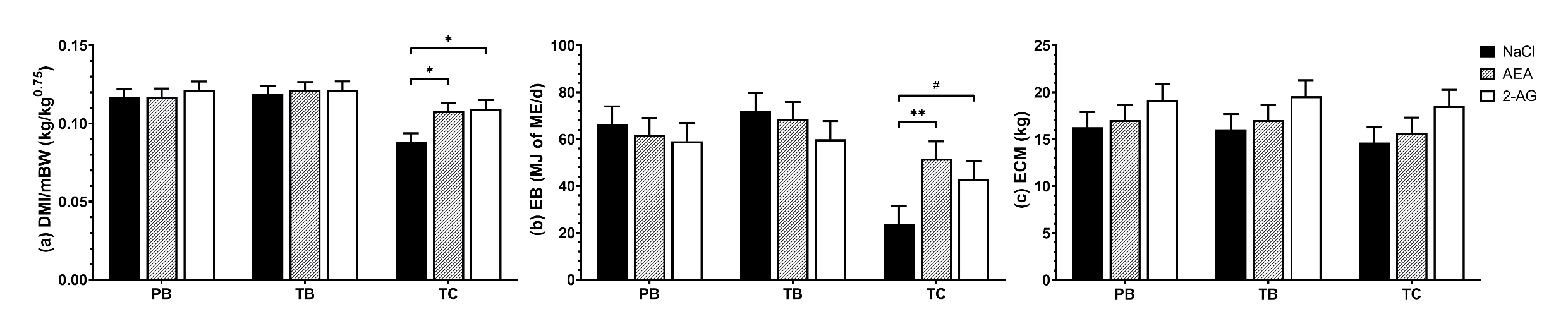


**Supplementary Figure S2.** Dry matter intake (DMI) per mBW (a), energy balance (EB) (b) and energy corrected milk yield (ECM) (c) pre-treatment (PB), after intraperitoneal administration with NaCl (n = 7), AEA (n = 7) or 2-AG (n = 6) under normal, non-stressed conditions in the barn (TB), and under stressed conditions in the respiration chamber (TC), respectively. DMI/mBW, EB and ECM did not differ between tratment groups PB and TB and pairwise effects were detected only for TC for DMI/mBW and EB, with the control group having lower DMI/mBW and EB compared to AEA and 2-AG treatment. Graphs are presented as highest level of significant interaction, associated *P*-values can be found in Supplementary Table S3. Within-housing differences are indicated by # *P* < 0.1, * *P* < 0.05 and ** *P* < 0.01 (Tukey-Kramer).

| **Supplementary Table S1.** Ingredients and chemical composition (means ± SE) of the grass silage (GS) and corn silage (CS) based total mixed ration (TMR) | | |
| --- | --- | --- |
|  |  |  |
| Component | GS | CS |
| Ingredient, g/kg of DM |  |  |
| Grass silage | 464.9 ± 18.1 |  |
| Corn silage |  | 570.9 ± 7.3 |
| Barley straw |  | 57.7 ± 13.9 |
| Grass hey | 131.9 ± 17.1 |  |
| Corn, ground | 114.9 ± 18.9 | 78.7 ± 11.0 |
| Wheat, ground | 116.5 ± 1.5 | 44.4 ± 0.5 |
| Extracted soy meal |  | 95.8 ± 5.2 |
| Extracted rapeseed meal |  | 134.8 ± 1.7 |
| Concentrate^1^ | 114.4 ± 5.1 |  |
| Mineral/vitamin mix^2^ | 7.4 ± 0.1 | 9.9 ± 0.1 |
| Lime stone powder^3^ |  | 5.1 ± 0.1 |
| Animal feed salt^4^ |  | 2.7 ± 0.0 |
| Chemical analysis, g/kg of DM |  |  |
| Crude ash | 76.4 ± 2.1 | 40.6 ± 2.3 |
| Crude protein | 161.6 ± 3.9 | 158.0 ± 3.5 |
| Crude fiber | 190.8 ± 7.9 | 164.0 ± 2.3 |
| Crude fat | 28.0 ± 1.4 | 34.0 ± 0.7 |
| Starch | 184.2 ± 20.4 | 298.0 ± 7.3 |
| aNDFom | 471.4 ± 17.3 | 385.4 ± 5.3 |
| ADFom | 235.2 ± 11.1 | 198.8 ± 3.5 |
| ME (MJ/kg of DM)^5^ | 10.7 ± 0.2 | 11.5 ± 0.1 |
| NE_L_ (MJ/kg of DM)^5^ | 6.5 ± 0.1 | 7.0 ± 0.1 |
| Fatty acid proportions (% of total lipids) |  |  |
| C16:0 | 17.5 ± 0.7 | 14.3 ± 0.2 |
| C18:0 | 2.0 ± 0.1 | 2.1 ± 0.1 |
| C18:1cis9 | 11.8 ± 1.1 | 24.9 ± 0.5 |
| C18:2n6 | 30.7 ± 1.3 | 43.6 ± 0.8 |
| C18:3n3 | 32.3 ± 3.3 | 8.3 ± 0.5 |
| n-6/n-3 ratio | 1.03 ± 0.13 | 5.55 ± 0.38 |
| ^1^MF 2000 (Ceravis AG, Regensburg, Germany): 30% extracted soy meal from peeled and steam-heated beans, 25.4% corn grit, 15% malt culms, 6.9% extracted rapeseed meal, 5.1% wheat, 5% sugar beet pulp, 4.9% dried grain pulp, 4.1% beet molasses, 2% NaHCO_3_, 1% CaCO_3_, 0.2% NaCl. Additives per kg original substance: 10,000 IU vitamin A, 1125 IU vitamin D_3_, 40 mg vitamin E, 0.6 mg I , 0.4 mg Co , 50 mg Mn, 75 mg Zn, 0.4 mg Se. Composition: 24% CP, 2.6% crude fat, 5.1% crude fiber, 8% crude ash, 0.73% Ca, 0.5% P, 0.65% Na, 7.1 MJ NEL/kg. | | |
| ^2^Panto®-Mineral R 8609 (HL Hamburger Leistungsfutter GmbH, Hamburg, Germany): 32.8% CaNaPO_3_, 19.3% CaMg(CO₃)₂, 16.7% NaCl, 15.3% CaCO_3_, 6.1% MgO, 3% sugar cane molasses. Additives per kg original substance: 900,000 IU vitamin A, 200,000 IU vitamin D_3_, 4.5 g vitamin E, 1.5 g Cu, 8 g Zn, 5 g Mn, 60 mg I, 70 mg Co, 50 mg Se. Composition: 20% Ca, 6% P, 8% Na, 6% Mg. | | |
| ^3^Kreidekalk (Spezialfutter Neuruppin GmbH und Co.KG): CaCO_3_. Composition: 37% Ca. | | |
| ^4^ESCO Viehsalz (esco - european salt company GmbH, Hannover, Germany) Composition: 99% NaCl, 0.3% Ca, 0.01% Mg, <2 mg/kg Cu, < 2 mg/kg Zn | | |
| ^5^ME and NEL content of TMR were calculated according to the recommendations of the German Society of Nutrition Physiology (GfE). | | |

| **Supplementary Table S2.** Primer sequences | | | | | | |
| --- | --- | --- | --- | --- | --- | --- |
| Gene |  | Primer sequence (5’ to 3’) | Accession no. | bp^1^ | Annealing  (°C / s) | Efficiency |
|  |  |  |  |  |  |  |
| ACAA2 | fwd | TGAATGAAGCTTTTGCTCCTC | NM_001035342.2 | 224 | 60 | 1.85 |
|  | rev | GATGATGACCGCAATTCCTT |  |  |  |  |
| ACACA | fwd | CTCTTCCGACAGGTTCAAGC | NM_174224.2 | 248 | 60 | 1.88 |
|  | rev | ACCATCCTGGCAAGTTTCAC |  |  |  |  |
| CNR1 | fwd | AAGCCCGCATGGACATTCGGCTGG | NM_001242341.2 | 79 | 60 | 1.88 |
|  | rev | AGCAGAGGGCCCCAGCAGAT |  |  |  |  |
| CPT1A | fwd | GGGTCACCTTTGGGAGTACG | NM_001304989.2 | 141 | 60 | 1.82 |
|  | rev | TCCTGACATTCCTCCGGGAT |  |  |  |  |
| DGAT1 | fwd | GACCCCTAACCTTTGACCCC | NM_174693.2 | 163 | 60 | 1.82 |
|  | rev | CCCAACCTCCCGCTAAGTTT |  |  |  |  |
| DGAT2 | fwd | GAAAGGTAGGAGCACGGGTC | NM_205793.2 | 132 | 60 | 1.84 |
|  | rev | AGCCCAACACTATTCACGCA |  |  |  |  |
| EIE3K | fwd | CCAGGCCCACCAAGAAGAA | NM_001034489 | 125 | 60 | 1.84 |
|  | rev | TTATACCTTCCAGGAGGTCCATGT |  |  |  |  |
| FAAH | fwd | TTCCTGCCAAGCAACATACCT | NM_001099102.2 | 105 | 60 | 1.86 |
|  | rev | CACGAAATCACCTTTGAAGTTCTG |  |  |  |  |
| GRP55 | fwd | TGCTGCCTGGATGTTTTCTG | XM_024982054.1 | 207 | 60 | 1.88 |
|  | rev | TCAGCCCACCACATCAGG |  |  |  |  |
| HADH | fwd | AACACCAATGACCAGCCAGA | NM_001046334.1 | 150 | 60 | 1.88 |
|  | rev | TCACACAGCCTGACTGCTTC |  |  |  |  |
| PLAAT5 | fwd | AAGATCATCCAGCGGACAAAA | NM_001281908.2 | 100 | 60 | 1.84 |
|  | rev | CGCCGTATCTCAGGTCATTGA |  |  |  |  |
| MGLL | fwd | CTTCTCTGTGGGTCTTCGCT | NM_001206681.1 | 137 | 62 | 1.91 |
|  | rev | TCCGTCTTATTCCTCGAGAGC |  |  |  |  |
| PPARA | fwd | CGGAAGTCCGCATTTTCCAC | NM_001034036.1 | 171 | 60 | 1.85 |
|  | rev | TCACAGAAGACAGCATCGCA |  |  |  |  |
| PPIA | fwd | GGATTTATGTGCCAGGGTGGTGA | NM_178320 | 120 | 60 | 1.90 |
|  | rev | CAAGATGCCAGGACCTGTATG |  |  |  |  |
| SREBF1 | fwd | GCACCGAGGCCAAGTTGAAT | NM_001113302.1 | 149 | 60 | 1.92 |
|  | rev | CACCAGTCCTTCAGCGATT |  |  |  |  |
| ^1^Product size (base pairs)  ACAA2, acetyl-COA acyltransferase 2; ACACA, acetyl-CoA carboxylase alpha; CNR1, cannabinoid receptor 1; CPT1A, carnitine palmitoyltransferase 1A; DGAT1/ 2, diacylglycerol O-acyltransferase 1/ 2; EIE3K, eukaryotic translation initiation factor 3 subunit K; FAAH, fatty acid amide hydrolase; GPR55, G protein-coupled receptor 55; HADH, hydroxyacyl-CoA dehydrogenase; PLAAT5, phospholipase A and acyltransferase 5; MGLL, monoglyceride lipase; PPARA, peroxisome proliferator activated receptor alpha; PPIA, Peptidylprolyl isomerase A; SREBF1, sterol regulatory element binding transcription factor 1 | | | | | | |

| **Supplementary Table S3.** *P*-values of the effects of treatment, diet, housing/ interval and their interactions using the MIXED procedure in SAS (SAS Institute, Cary, NC, USA) | | | | | | | |
| --- | --- | --- | --- | --- | --- | --- | --- |
|  | *P*-value | | | | | | |
|  | Treatment | Diet | Housing | Treatment x Housing | Treatment x Diet | Diet x Housing | Treatment x Diet x Housing |
| Fig. 1 A Dry Matter Intake (DMI) per mBW change (%) | 0.08 | 0.79 | < 0.001 | < 0.01 | 0.61 | 0.79 | 0.29 |
| Fig. 1 B Energy Balance (EB) change (%) | < 0.05 | 0.44 | < 0.001 | < 0.05 | 0.37 | 0.12 | 0.34 |
| Fig. 1 C Energy corrected milk yield (ECM) change (%) | 0.06 | 0.35 | < 0.001 | 0.61 | 0.81 | 0.61 | 0.45 |
|  |  |  |  |  |  |  |  |
|  | Treatment | Diet | Housing | Treatment x Housing | Treatment x Diet | Diet x Housing | Treatment x Diet x Housing |
| Fig. S2 A Dry Matter Intake (DMI) per mBW | 0.36 | < 0.05 | < 0.001 | < 0.001 | 0.92 | 0.76 | 0.32 |
| Fig. S2 B Energy Balance (EB) | 0.61 | < 0.05 | < 0.001 | < 0.001 | 0.79 | 0.70 | 0.20 |
| Fig. S2 C Energy corrected milk yield (ECM) | 0.26 | < 0.05 | < 0.001 | 0.64 | 0.82 | 0.64 | 0.73 |
|  |  |  |  |  |  |  |  |
|  | Treatment | Diet |  |  | Treatment x Diet |  |  |
| Fig. 2 A NEFA change (%) | < 0.001 | 0.42 |  |  | 0.25 |  |  |
| Fig. 2 B Triglyceride change (%) | 0.50 | 0.06 |  |  | 0.59 |  |  |
| Fig. 2 C Cholesterol change (%) | 0.72 | 0.32 |  |  | < 0.05 |  |  |
|  |  |  |  |  |  |  |  |
|  | Treatment | Diet | Interval | Treatment x Interval | Treatment x Diet | Diet x Interval | Treatment x Diet x Interval |
| Fig. 3 A Hourly Dry Matter Intake (DMI) per mBW | < 0.05 | 0.12 | < 0.001 | < 0.05 | 0.83 | < 0.001 | 0.81 |
| Fig. 3 B Cumulative Dry Matter Intake (DMI) per mBW | 0.05 | 0.14 | < 0.001 | < 0.001 | 0.76 | < 0.001 | 1.00 |
| Fig. 3 C Changes in Fat Oxidation per mBW (Δ FOX/mBW) | 0.21 | < 0.05 | < 0.001 | 0.10 | 0.62 | < 0.001 | 0.51 |
| Fig. 3 D Changes in Carbohydrate Oxidation per mBW (Δ COX/mBW) | < 0.01 | 0.55 | < 0.001 | < 0.01 | 0.34 | < 0.001 | 0.47 |
| Fig. 3 E Changes in Heat Production per mBW (Δ HP/mBW) | < 0.01 | < 0.001 | < 0.001 | < 0.001 | 0.48 | < 0.001 | 0.81 |
| Fig. 3 F Mean daily FOX/(DMI x mBW) | 0.08 | 0.09 |  |  | 0.52 |  |  |
| Fig. 3 G Mean daily COX/(DMI x mBW) | 0.30 | 0.05 |  |  | 0.24 |  |  |
| Fig. 3 H Mean daily HP/(DMI x mBW) | 0.25 | 0.33 |  |  | 0.64 |  |  |

| **Supplementary Table S4.** Percent changes (%) of major plasma fatty acids in total lipids from pre-treatment (PB) to after intraperitoneal administration with NaCl (n = 7), AEA (n = 7) or 2-AG (n = 6). The individual percent changes were calculated and tested by ANOVA. Cows were fed ad libitum a grass silage (GS) and corn silage (CS) based diet. | | | | | | | | | | | | |
| --- | --- | --- | --- | --- | --- | --- | --- | --- | --- | --- | --- | --- |
|  | GS | | |  | CS | | |  | *P*-value | | |  |
|  | NaCl | AEA | 2AG |  | NaCl | AEA | 2AG |  | Treatment | Diet | Treatment x diet |  |
| C16:0 | 3.74 ± 2.72 | -2.02 ± 2.72 | -1.26 ± 2.91 |  | 6.97 ± 2.72 | -5.86 ± 2.72 | -3.23 ± 2.91 |  | <0.01 | 0.73 | 0.31 |  |
| C16:1 | 5.33 ± 3.13 | -7.12 ± 3.13 | -7.18 ± 3.34 |  | -2.89 ± 3.13 | -7.46 ± 3.13 | -6.54 ± 3.34 |  | <0.05 | 0.29 | 0.27 |  |
| C18:0 | -3.14 ± 3.16 | 2.88 ± 3.16 | 3.13 ± 3.35 |  | 0.51 ± 3.16 | 4.78 ± 3.16 | 6.61 ± 3.35 |  | 0.09 | 0.37 | 0.94 |  |
| C18:1c9 | 20.7 ± 5.07 | 0.99 ± 5.07 | 2.36 ± 5.47 |  | 9.86 ± 5.07 | -3.45 ± 5.07 | -5.49 ± 5.47 |  | <0.01 | 0.07 | 0.79 |  |
| C18:2n6 | -3.83 ± 1.60 | 0.41 ± 1.60 | -1.05 ± 1.69 |  | -4.20 ± 1.60 | 2.41 ± 1.60 | -0.16 ± 1.69 |  | <0.01 | 0.47 | 0.69 |  |
| C18:3n3 | 3.92 ± 3.94 | 2.51 ± 3.94 | -0.51 ± 4.05 |  | -7.51 ± 3.94 | -0.85 ± 3.94 | -3.67 ± 4.05 |  | 0.31 | 0.26 | 0.28 |  |
| C20:2n6 | -7.14 ± 8.60 | 0.01 ± 8.60 | 0.74 ± 9.28 |  | 10.36 ± 8.6 | 11.47 ± 8.6 | 2.51 ± 9.28 |  | 0.91 | 0.09 | 0.43 |  |
| C20:3n6 | 0.21 ± 4.00 | 3.14 ± 4.00 | 8.88 ± 4.31 |  | 6.68 ± 4.00 | 10.8 ± 4.00 | 15.4 ± 4.31 |  | 0.20 | 0.06 | 0.98 |  |
| C20:4n6 | 2.58 ± 5.23 | -3.04 ± 5.23 | -1.65 ± 5.45 |  | 17.2 ± 5.23 | 11.3 ± 5.23 | 9.90 ± 5.45 |  | 0.33 | 0.05 | 0.90 |  |
| C20:5n3 | 12.6 ± 4.86 | 7.28 ± 4.86 | 11.4 ± 5.09 |  | 13.3 ± 4.86 | 2.08 ± 4.86 | -3.81 ± 5.09 |  | 0.05 | 0.26 | 0.18 |  |
| C22:4n6 | 2.68 ± 9.45 | 0.62 ± 9.45 | 8.57 ± 9.85 |  | 25.2 ± 9.45 | 25.6 ± 9.45 | 23.8 ± 9.85 |  | 0.93 | 0.08 | 0.70 |  |
| C22:5n3 | 17.03 ± 9.53 | 7.77 ± 9.53 | 19.7 ± 9.96 |  | 24.22 ± 9.53 | 12.8 ± 9.53 | 8.46 ± 9.96 |  | 0.53 | 0.97 | 0.17 |  |
| C22:6n3 | 38.8 ± 23.2 | 51.5 ± 23.2 | 24.2 ± 24.7 |  | 36.9 ± 23.2 | -2.34 ± 23.2 | 25.2 ± 24.7 |  | 0.62 | 0.40 | 0.50 |  |
| n-6/n-3 ratio | -8.72 ± 5.11 | -2.63 ± 5.11 | -1.55 ± 5.24 |  | -1.92 ± 5.11 | 3.03 ± 5.11 | 4.28 ± 5.24 |  | 0.05 | 0.38 | 0.98 |  |
